# Umbilical Artery Doppler and Adverse Outcomes in Severe Preeclampsia Without Fetal Growth Restriction: A Retrospective Cohort Study

**DOI:** 10.7759/cureus.67850

**Published:** 2024-08-26

**Authors:** Daniel Core, Dani Zoorob, Rose Maxwell, Madison Catalanotto Maas, Elizabeth Hixson Richardson, David Fucinari, Christopher Menefee, Layne Landry, Perry Barrilleaux

**Affiliations:** 1 Obstetrics and Gynecology, Louisiana State University Health Sciences Center Shreveport, Shreveport, USA; 2 Obstetrics and Gynecology, Wright State University, Dayton, USA

**Keywords:** obstetric ultrasound, hypertension in pregnancy, fetal outcomes, umbilical artery doppler, preeclampsia

## Abstract

Background and objective

Severe preeclampsia may be managed expectantly before 34 weeks gestation with close surveillance. Utilized in fetal growth restriction (FGR), evidence supports umbilical artery (UA) Doppler preventing neonatal morbidity from hypertensive disease and predicting adverse outcomes in preeclampsia.

We evaluated the association of abnormal UA Doppler waveforms with early delivery (before 34 weeks gestation) and adverse maternal-fetal outcomes in patients with early severe preeclampsia without FGR.

Methodology

This is a retrospective cohort study of singleton pregnancies with International Classification of Diseases (ICD) Ninth or Tenth Revision, defined severe preeclampsia diagnosed before 34 weeks gestation without FGR from January 1, 2018, through January 27, 2023, at a large tertiary care center where S/D ratios were calculated from UA Doppler interrogation of a free loop of cord at least once weekly. This study was approved by the IRB (ID:00002216) and granted a full Health Insurance Portability and Accountability Act (HIPAA) waiver of consent. Exclusion criteria were major congenital anomalies, congenital infection, aneuploidy, leaving against medical advice >24 hours, and patient instability on admission defined as condition(s) precluding expectant management by the American College of Obstetrics and Gynecology. The primary outcome was delivery before 34 weeks gestation. Secondary outcomes were the mode of delivery and maternal/fetal complications.

Patient characteristics and outcomes for normal versus abnormal UA Doppler groups were compared with chi-square, t-tests, and Fisher’s exact test. Odds ratios and relative risks were calculated to compare outcomes.

Results

Of 194 patients with severe preeclampsia, 107 met inclusion criteria. Thirty-four patients had abnormal UA Doppler studies. There were no differences in demographic and clinical data between patients with normal and abnormal UA Doppler studies. Patients with abnormal UA Doppler studies were more likely to deliver before 34 weeks (OR=3.91; 95% CI 1.24-12.33) for worsening severe features (OR=3.85; 95% CI 1.42-10.41), and were less likely to deliver vaginally (OR=0.12; 95% CI 0.03-0.54). Abnormal UA Doppler studies were associated with an increased risk of neonatal complications (OR=6.46; 95% CI 1.42-29.42) and respiratory distress syndrome (RDS) (OR=4.75; 95% CI 1.32-17.16). Abnormal UA Doppler subgroups were divided into patients with elevated S/D >95% Acharya (N=22) and absent end-diastolic flow (EDF) (N=10). The elevated S/D group tended to deliver before 34 weeks gestation for worsening severe features (OR=3.71, 95% CI 1.144-12.050) and had a higher risk of neonatal complications (RR 1.404; 95% CI 1.213-1.624). The absent EDF subgroup was more likely to deliver before 34 weeks (RR=1.52; 95% CI 1.29-1.79) for abnormal fetal testing (OR=6.92; 95% CI 1.71-28.08) and undergo primary cesarean delivery (OR=7.23; 95% CI 1.43-36.61).

Conclusion

Pregnancies with severe preeclampsia without FGR displayed a high incidence of abnormal UA Doppler waveforms associated with loss of clinical stability and adverse fetal outcomes. The groups with more impedance to umbilical artery flow tended to deliver earlier, and as the Doppler shifted from elevated S/D to absent end-diastolic flow, the mode of delivery shifted to cesarean delivery with increased risk of abnormal fetal testing. These results support the utility of UA Doppler surveillance in severe preeclampsia.

## Introduction

Preeclampsia is a complex disorder with large phenotypic variation broadly characterized as elevated blood pressure at or beyond 20 weeks gestation with elevated proteinuria and/or evidence of organ dysfunction [1]. It affects 2-8% of pregnancies [2] and is a major contributor to maternal mortality worldwide [2]. Criteria for severe preeclampsia vary by professional society [3-7], but it is broadly defined as new-onset severe hypertension after 20 weeks of gestation with or without evidence of end-organ damage.

Early severe preeclampsia (diagnosed <34 weeks gestation) is associated with greater morbidity than when the disorder presents at term [5,8,9]. Early severe preeclampsia is a disease of placental origin characterized by malplacentation and unremodelled spiral arteries in the first half of pregnancy [7]. The small muscular spiral arteries fail to dilate and lose their endothelium, smooth muscle, and inner elastic lamina in the second trimester, as occurs in normal pregnancy [10]. This failed remodeling is reflected in an increased incidence of a persistent diastolic notch in uterine artery Doppler waveforms after 22-24 weeks of gestation [11,12]. In contrast, late-onset preeclampsia is characterized by placental compression and hypoxia later in pregnancy. A 2013 comparative study of 456,668 singleton pregnancies showed a 0.3% incidence of early-onset severe preeclampsia and that early preeclampsia was more strongly associated with chronic hypertension and worse fetal outcomes compared to late-onset disease [8]. This cohort was expanded in 2014 to include 670,120 singleton gestations, which revealed worse maternal morbidity with early severe preeclampsia [9]. These distinctions support recommendations that severe preeclampsia be divided into two groups by time of onset because of differences in prognosis and management [12].

In the United States, early severe preeclampsia may be managed expectantly if maternal-fetal stability is established up to 34 weeks gestation with close surveillance [7,13,14]. There is no high-quality data for specific frequency and/or type of fetal surveillance [14]. Umbilical artery (UA) Doppler evaluation is used to monitor for patency of fetoplacental blood flow and is a recommended component of fetal surveillance in pregnancies affected by fetal growth restriction (FGR) and twin-twin transfusion syndrome [15,16]. Progression of fetoplacental circulatory impedance in high-risk pregnancies is reflected by diminishing end-diastolic flow (EDF) in the UA and a rise in the UA Doppler indices [17]. Evidence supports UA Doppler preventing neonatal morbidity from hypertensive disease [18] and predicting adverse outcomes in preeclampsia [19]. The Society for Maternal Fetal Medicine (SMFM), the American College of Obstetricians and Gynecologists (ACOG), the International Society for the Study of Hypertension in Pregnancy (ISSHP), and the National Institute for Health and Care Excellence (NICE) list reversal of diastolic flow as an indication for delivery in severe preeclampsia, but only NICE currently recommends evaluating and surveillance of umbilical artery Doppler flow [13,14,20,21].

At our institution, all expectantly managed cases of severe preeclampsia undergo at least twice daily non-stress tests and weekly biophysical profiles with UA Doppler interrogation. We evaluated the association of abnormal UA Doppler waveforms with early delivery prior to 34 weeks and indication for delivery and adverse maternal-fetal outcomes in patients with early severe preeclampsia without FGR. Preliminary results of this study were presented at the American College of Obstetrics and Gynecology (ACOG) District VII Annual Meeting, 09/28/23 - 09/30/23, Tulsa, OK.

## Materials and methods

This study was conducted at Louisiana State University Health Shreveport and approved by the Institutional Review Board (IRB ID: STUDY00002216) (DHHS Federal Wide Assurance Identifier FWA 00000653). Using the Splicer Dicer chart retrieval feature of the Epic medical record system, all singleton gestations delivered between January 1, 2018 through January 27, 2023 within the LSU Health-Shreveport Hospital system with International Classification of Diseases, Ninth/Tenth Revision (ICD-9/10) codes for severe preeclampsia (O14.1); severe preeclampsia, third trimester (O14.13); severe preeclampsia, second trimester (O14.12); and pre-existing hypertension with preeclampsia (O11) which had a delivery date at or before 34 weeks gestation were retrieved. All charts containing the code for intrauterine growth restriction (O36.5) and twin pregnancy (O30.0) were filtered out during chart retrieval.

Exclusion criteria were multiple gestations, major congenital anomalies, congenital infection, aneuploidy, if the patient left against medical advice for greater than 24 hours, and if the patient was unstable and delivered upon admission. The primary outcome was delivery prior to 34 weeks gestation along with the indication for delivery. Secondary outcomes include primary cesarean delivery, cesarean delivery due to fetal distress, vaginal delivery, and maternal and fetal complications. Each patient’s chart was individually reviewed by at least two members of the study team to confirm inclusion and exclusion criteria were met, to review the Doppler measurements, and to identify the outcomes and indications for delivery.

Fetal growth restriction was defined as estimated fetal weight less than the tenth percentile Hadlock [22] or Alexander [23] or by abdominal circumference less than the tenth percentile Hadlock [22] per ACOG and SMFM guidelines. Abdominal circumference was not used prior to February 2021, when ACOG and the SMFM updated their definition of FGR. UA Doppler interrogations were performed at least once weekly. Systolic/diastolic ratios (S/D) were calculated from Doppler interrogation of a free loop of the umbilical cord in the absence of fetal breathing or movement with 1-3 Doppler measurements taken. Elevated S/D is defined as S/D >95th percentile Acharya [24]. If intermittent absence or reversal was encountered, it was counted as such if it was obtained and the other measurements were abnormal. For instance, if “intermittent absent diastolic flow” was documented or a single instance of absent EDF was documented with the other measures within two weeks of the absent end diastolic flow (EDF) being elevated S/D, the patient was recorded as having absent EDF. If only one instance of absence or reversal was encountered in one segment of the cord and all other measurements were normal, this was considered to be an artifact, and these patients were not treated as if they had absent/reversed EDF by clinicians once it was ruled out with a repeat Doppler interrogation within 48 hours and did not recur for the remainder of the hospital course.

Race was self-reported with a standardized institutional questionnaire. The labels White, Black, and None of the above were used due to the majority of patients in the cohort being Black or White. Three patients were categorized as None of the above and were self-reported as Hispanic/Other (N=1), Asian (N=1), and American Indian (N=1). Other medical-demographic variables collected were age, ethnicity (Hispanic vs. non-Hispanic), insurance carrier (Medicaid, private, other), primiparity, body mass index (BMI) (<20, 20-29.9, 30-39.9, 40-49.9, 50 or greater), chronic hypertension, history of severe preeclampsia, diabetes, autoimmune disease, and thrombophilia.

Indications for delivery prior to 34 weeks were equivocal fetal testing defined as abnormal non-stress test and/or biophysical profile, preterm labor, worsening severe feature, or other. Worsening severe features included headache unrelieved by medication(s), severe range blood pressures requiring multiple fast-acting anti-hypertensive medication administrations, persistent visual disturbance, epigastric pain or right upper pain unresponsive to repeat analgesics, hemolysis, elevated liver enzymes, low platelet count (HELLP) syndrome, new or worsening renal dysfunction (serum creatinine greater than 1.1 mg/dL or twice baseline), pulmonary edema, eclampsia, or vaginal bleeding in the absence of placenta previa.

Maternal outcomes were HELLP (defined as transaminase values twice normal concentration with platelet count less than 100,000 plt/L and an upward trend in lactate dehydrogenase), neurologic manifestation (defined as seizure, stroke, or posterior reversible encephalopathy syndrome (PRES)), renal failure (creatinine greater than 1.1 mg/dL or twice baseline), liver transaminase values twice normal concentration, pulmonary edema, or death. Fetal complications included acidosis (umbilical artery pH < 7.1), five-minute appearance, pulse, grimace, activity, and respiration (APGAR) <7, perinatal death, sepsis (blood culture positive), IVH (grade III or less), periventricular leukomalacia, respiratory distress syndrome (defined as need for ventilatory support and pathologic findings on chest imaging), bronchopulmonary dysplasia, and necrotizing enterocolitis.

Patient characteristics and outcomes for patient groups with normal versus abnormal results were compared with chi-square, t-tests, and Fisher’s exact test where appropriate. Odds ratios and relative risks were calculated to compare outcomes. Statistics were calculated using IBM SPSS Statistics for Windows, Version 29 (Released 2023; IBM Corp., Armonk, New York, United States).

## Results

**Figure 1:**
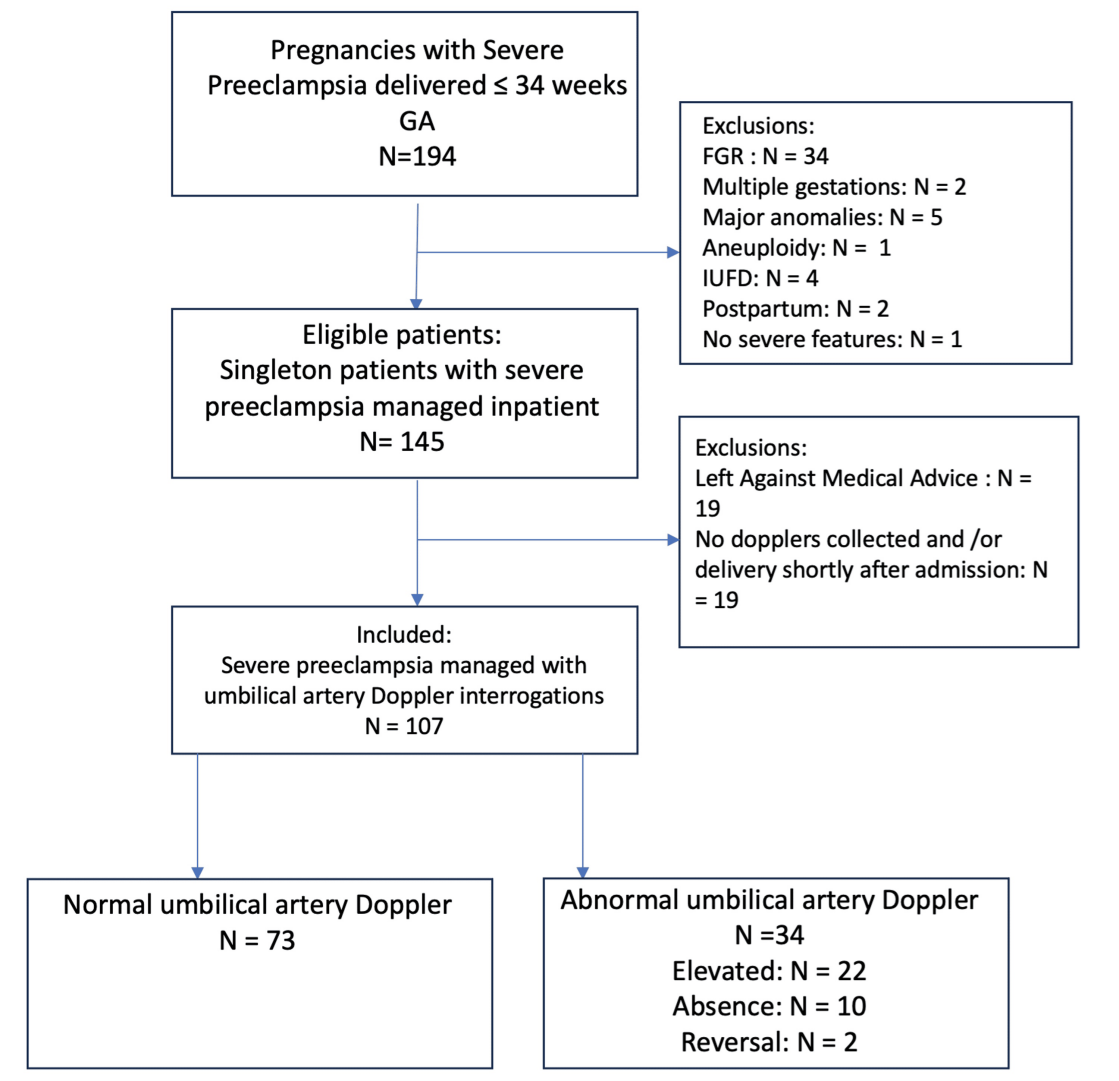
Study inclusion decision tree IUFD: intrauterine fetal demise; GA: gestational age; FGR: fetal growth restriction

Of 194 patients with severe preeclampsia, 107 met inclusion criteria (Figure 1). There were no differences in demographic and clinical data between normal (N=73) and abnormal UA Doppler (N=34) groups. The abnormal UA Doppler group was divided into three subgroups: elevated (N=22), absence (N=10), and reversal (N=2). The outcomes of patients with abnormal UA Doppler studies differed significantly from those with normal UA Doppler studies (Table 1). Patients in the abnormal UA Doppler group developed severe preeclampsia 1.9 weeks earlier (95%, P<.001) at 29.4 weeks gestation (±2.8 weeks) and delivered 1.4 weeks earlier (95%, P<.001) than those with normal UA Doppler studies at mean 30.9 weeks gestation (±2.6 weeks). The primary outcome of delivering prior to 34 weeks gestation was significantly different between the two groups. Patients with abnormal Dopplers were more likely to deliver before 34 weeks (odds ratio (OR)=3.91; 95% confidence interval (CI) 1.24-12.33) due to worsening severe features (OR=3.85; 95% CI 1.42-10.41) and were less likely to deliver vaginally (OR=0.12; 95% CI 0.03-0.54). The development of abnormal UA Doppler increased the risk of composite neonatal complications (OR=6.46; 95% CI 1.42-29.42) and respiratory distress syndrome (OR=4.75; 95% CI 1.32-17.16). Of patients with abnormal Doppler studies, 35.3% had a maternal complication compared to 19.2% of patients with normal Dopplers, although this did not reach statistical significance (OR=2.30, 95% CI 0.92-5.73).

**Table 1:** Outcomes by Doppler result GA: gestational age; HELLP: hemolysis, elevated liver enzymes and low platelets syndrome; RR: relative risk; T: T-test; APGAR: appearance, pulse, grimace, activity and respiration a: presented as Mean (±SD); b: presented as N (%) * indicates statistical significance (P<.05 or CI does not include 1.0)

	Normal	Abnormal	T-test (P) or Odds Ratio/Relative Risk(CI)
	N = 73 (68.2%)	N= 34 (31.8%)	
GA at diagnosis of severe preeclampsia (weeks)* ^a^	31.19 (±1.792)	29.35(±2.751)	P < .001 (T)
GA at delivery (weeks)* ^a^	32.3 (±1.7)	30.9 (±2.6)	P < .001 (T)
Birth weight (grams)* ^a^	1950.0 (±417.7)	1590.0 (±543.7)	P < .001 (T)
Delivered before 34 weeks* ^b^	48 (65.8%)	30 (88.2%)	3.91 (1.24 – 12.33)
Indication for delivery: fetal testing ^b^	13 (17.8%)	11 (32.4%)	2.21 (0.87 – 5.63)
Indication for delivery: worsening severe feature* ^b^	40 (54.8%)	28 (82.4%)	3.85 (1.42 – 10.41)
Indication for delivery: preterm labor ^b^	3 (4.1%)	0	RR 0.30 (0.02 – 5.69)
Indication for delivery: other ^b^	5 (6.8%)	1 (2.9%)	0.412 (0.05 – 3.67)
Vaginal delivery* ^b^	25 (34.2%)	2 (5.9%)	0.12 (0.03 - 0.54)
Primary cesarean delivery ^b^	26 (35.6%)	15 (44.1%)	1.43 (0.62 – 3.27)
Cesarean delivery due to fetal distress ^b^	11 (15.1%)	2 (5.9%)	0.35 (0.07 – 1.69)
Maternal complication ^b^	14 (19.2%)	12 (35.3%)	2.30 (0.92 – 5.73)
HELLP ^b^	1 (1.4%)	1 (2.9%)	2.18 (0.13 – 35.96)
Neurologic manifestation ^b^	0	2 (5.7%)	RR 10.57 (0.52 – 214.40)
Renal failure ^b^	4 (5.5%)	5 (14.7%)	2.97 (0.75 – 11.88)
Pulmonary edema ^b^	4 (5.5%)	1 (2.9%)	0.52 (0.06 – 4.86)
Transaminitis ^b^	7 (9.6%)	4 (11.8%)	1.26 (0.34 – 4.62)
Maternal death ^b^	0	0	-
ICU admission ^b^	5 (6.8%)	5 (14.7%)	2.35 (0.63 – 8.72)
Neonatal complication* ^b^	52 (71.2%)	32 (94.1%)	6.462 (1.4219 – 29.4219)
Acidosis (pH <7.1) ^b^	3 (4.1%)	0	RR 0.30 (0.02 – 5.69)
5 minute APGAR < 7 ^b^	3 (4.1%)	5 (14.7%)	4.02 (0.90 – 17.95)
Perinatal death ^b^	0	0	-
Neonatal sepsis ^b^	1 (1.4%)	0	RR 0.70 (0.03 – 16.87)
Intraventricular hemorrhage (grade III or less) ^b^	5 (6.8%)	6 (17.6%)	2.91 (0.82 – 10.33)
Periventricular leukomalacia ^b^	7 (9.6%)	2 (5.9%)	0.59 (0.12 – 3.00)
Respiratory distress syndrome* ^b^	50 (68.5%)	31 (91.2%)	4.75 (1.32 – 17.16)
Bronchopulmonary dysplasia ^b^	1 (1.4%)	3 (8.8%)	6.34 (0.27 – 151.81)
Necrotizing enterocolitis ^b^	0	1 (2.9%)	RR 6.08 (0.25 – 145.65)

Differentiating the abnormal Doppler group further into elevated S/D and absent EDF groups shows further differences in timing and indication of delivery (Table 2). Both groups developed severe preeclampsia earlier: 29.41 weeks GA (95%, P=.011) for the elevated S/D subgroup and 29.5 weeks (95%, P=.046) for the absent EDF group, compared to 31.4 weeks in the normal Doppler group. Both groups were delivered earlier, with mean gestational age at delivery 31.3 weeks (95%, p=.011) for the elevated S/D subgroup and at 30.3 weeks (95%, P=.032) for patients with absent EDF, compared to 32.3 weeks in patients with normal Doppler studies. The elevated S/D group tended to deliver before 34 weeks gestation for worsening severe features (OR=3.71, 95% CI 1.144-12.050) and were less likely to achieve vaginal delivery (OR=0.192, 95% CI .041-.888). Patients with elevated S/D had a higher risk of composite neonatal complications (RR 1.404, 95%, 1.213-1.624) and respiratory distress syndrome (OR 9.660, 95%, CI 1.224-76.246). Patients with absent EDF were more likely to deliver before 34 weeks (RR=1.52; 95% CI 1.29-1.79), 60% were delivered early for abnormal fetal testing (OR=6.92; 95% CI 1.71-28.08), and 80% underwent primary cesarean delivery (OR=7.23; 95% CI 1.43-36.61). None of the patients with absent EDF delivered vaginally.

**Table 2:** Outcomes by Doppler subgroup Elevated: subgroup of patients with umbilical artery S/D ratio >95%ile Acharya; Absence: subgroup of patients with absent end-diastolic-flow of the umbilical artery T: T-test; RR: relative risk; GA: gestational age; HELLP: hemolysis, elevated liver enzymes and low platelets syndrome; APGAR: appearance, pulse, grimace, activity and respiration a: presented as mean (±SD); b: presented as N (%) * indicates statistical significance (P<.05 or CI does not include 1.0)

	Elevated	T-test (P) or Odds Ratio/Relative Risk(CI)	Absence	T-test (P) or Odds Ratio/Relative Risk(CI)
	N= 22		N= 10	
GA at delivery* ^a ^(weeks)	31.27 (±2.354)	P = .011 (T)	30.30 (±3.020)	P = .032 (T)
GA at onset* ^a ^(weeks)	29.41 (±2.873)	P < .001 (T)	29.50 (±2.799)	P = .046 (T)
Delivered before 34 weeks ^b^	18 (81.8%)	2.344 (.716 – 7.676)	10 (100%)	RR 1.521 (1.289 – 1.795)
Indication for delivery: fetal testing* ^b^	4 (18.2%)	1.026 (.297 – 3.538)	6 (60%)	6.923 (1.707 – 28.076)
Indication for delivery: worsening severe features ^b^	16 (80%)	3.713 (1.144 – 12.050)	8 (80%)	3.300 (.655 – 16.618)
Indication for delivery: preterm labors ^b^	0	RR .457 (.025 – 8.574)	0	RR 0.961 (.053 – 17.375)
Indication for delivery: other ^b^	0	RR .293 (.017 – 5.092)	1 (10%)	1.511 (.158 – 14.435)
Vaginal deliverys* ^b^	2 (9.1%)	.192 (.041 – .888)	0	RR .132 (.009 – 2.015)
Primary CS* ^b^	6 (27.3%)	.678 (.236 – 1.944)	8 (80%)	7.231 (1.428 – 36.605)
CS d/t fetal distress ^b^	1 (4.5%)	.268 (.033 – 2.205)	1 (10%)	.626 (.072 – 5.448)
Maternal complication ^b^	8 (36.4%)	2.408 (.846 – 6.854)	3 (30%)	1.806 (.414 – 7.876)
HELLP ^b^	0	RR 1.073 (.045 – 25.438)	1 (10%)	8.000 (.459 – 139.290)
Neurologic manifestation ^b^	2 (9.1%)	RR 16.087 (.801 – 323.164)	0	-
Renal failure ^b^	4 (18.2%)	3.833 (.873 – 16.836)	1 (10%)	1.917 (.192 – 19.094)
Pulmonary edema ^b^	1 (4.5%)	.821 (.087 – 7.755)	0	RR 0.748 (.043 – 12.952)
Transaminitis ^b^	4 (18.2%)	2.095 (.552 – 7.958)	0	RR .449 (.028 – 7.315)
Death ^b^	0	-	0	-
ICU admission ^b^	2 (9.1%)	1.360 (.245 – 7.549)	2 (20%)	3.400 (.564 – 20.487)
Neonatal complication* ^b^	22 (100%)	RR 1.404 (1.213 – 1.624)	8 (80%)	1.615 (.316 – 8.247)
Acidosis (pH <7.1) ^b^	0	RR 0.460 (0.025 – 8.574)	0	RR 0.961 (.053 – 17.375)
5 minute APGAR < 7 ^b^	3 (13.6%)	3.684 (.688 – 19.742)	2 (20%)	5.833 (.844 – 40.307)
Perinatal death ^b^	0	-	0	-
Neonatal sepsis ^b^	0	RR 1.073 (.045 – 25.438)	0	RR 2.242 (.097 – 51.673)
Intraventricular hemorrhage (grade III or less) ^b^	4 (18.2%)	3.022 (.735 – 12.425)	2 (20%)	3.400 (.564 – 20.487)
Seizure ^b^	0	-	0	-
Periventricular leukomalacia ^b^	2 (9.1%)	.943 (.181 – 4.905)	0	.446 (.028 – 7.315)
Hypoxic-ischemic encehalopath ^b^	0	-	0	-
Respiratory distress syndrome* ^b^	21 (95.5%)	9.660 (1.224 – 76.246)	8 (80%)	1.840 (.362 – 9.356)
Bronchopulmonary dysplasia ^b^	2 (9.1%)	7.200 (.621 – 83.523)	1 (10%)	8.000 (.459 – 139.290)
Acute renal failure ^b^	0	-	0	-
Necrotizing enterocolitis ^b^	1 (4.5%)	RR 9.652 (.407 – 228.941)	0	-

## Discussion

This study demonstrates that pregnancies with severe preeclampsia without growth restriction display a high incidence of abnormal umbilical artery Doppler indices. We also demonstrate an association of abnormal umbilical artery flow in these pregnancies, with increased risk for loss of maternal-fetal stability necessitating early delivery, decreased chance of vaginal delivery, and adverse neonatal outcomes.

It is notable that there were no significant associations between demographic and medical variables with abnormal Doppler studies. This indicates that there may not be predictive factors for placental degeneration once early severe preeclampsia manifests. Perhaps once early severe preeclampsia manifests, the pathophysiology of preeclampsia supersedes other contributors to placental insufficiency such as diabetes and chronic hypertension. This finding supports the universal surveillance of umbilical artery EDF in these pregnancies due to the lack of significant medical associations with abnormal Doppler studies.

When the abnormal Doppler group was further analyzed, we found that the transition from elevated S/D indices to absent EDF was associated with even earlier delivery, more often for fetal indication, and an increased risk of primary cesarean delivery. The pregnancies with elevated S/D flow tended to be delivered due to worsening severe features, while the group with absent EDF tended to be delivered early due to abnormal fetal testing. This correlates with umbilical artery Doppler’s utility as a measure of impedance to blood flow, i.e., placental vascularity and function [25]. Thus, as placental reserve and umbilical artery flow deteriorate in these pregnancies, the loss of stability more often shifts to the fetus, leading to abnormal fetal testing, loss of tolerance for labor, and increased likelihood of primary cesarean delivery. In fact, all 10 patients with absent EDF were delivered by cesarean section, eight of which were primary cesarean deliveries. This finding supports offering patients with absent EDF cesarean delivery as in FGR [15] or at least counseling them on the high likelihood of cesarean delivery.

We were unable to find a study evaluating the utility of UA Dopplers in pregnancies with severe preeclampsia without growth restriction, making this study unique. FGR was previously listed as a severe feature of preeclampsia [26]. FGR and preeclampsia commonly occur separately, and newer studies demonstrate differing placental pathophysiology in these disorders [27]. However, both are marked by progressive placental compromise, particularly if diagnosed at early gestational age. In FGR, placental deterioration is reflected in the progression of abnormal Doppler waveforms: elevated to absent EDF to reversal of EDF and the progressively increased risk of stillbirth and neonatal morbidity [15]. It is feasible that this progressive placental deterioration also occurs in severe preeclampsia and would be characterized with progressive loss of EDF in serial umbilical artery Doppler studies. This study supports this by demonstrating progressively earlier delivery in groups with normal versus elevated S/D versus absent diastolic flow.

Our finding that abnormal Doppler studies of the umbilical artery are associated with early delivery due to worsening severe features suggests that UA Dopplers may be used to help predict loss of maternal stability in early preeclampsia. Inflammatory microparticles from necrosing syncytiotrophoblasts are thought to play a role in the maternal pathologic manifestations of preeclampsia [27]. These microparticles have been demonstrated to activate monocytes, affect coagulation, and damage the endothelium [27-29]. The association of worsening severe features with abnormal Doppler studies may be explained by increased release of inflammatory material causing vasospasm, organ damage, and/or coagulopathy as the placenta degrades, reflected by decreased EDF. This is a novel use of the umbilical artery Doppler, which has been traditionally used to predict neonatal morbidity.

Our study adds to the literature by demonstrating clinical utility in incorporating UA Doppler studies into fetal surveillance of pregnancies affected by early severe preeclampsia without FGR. Additional strengths include the large portion of classically underrepresented patient populations (Medicaid, Black) in our study cohort. Additionally, all clinical information was collected by a detailed review of the clinical documentation by two different researchers to ensure data integrity.

This study is limited due to its retrospective, unblinded nature. Our analysis relies on accurate documentation of subjective symptoms and blood pressure. It is possible that providers were biased towards earlier delivery when patients had abnormal Doppler studies. This potential bias would still be present in a prospective study due to the ethical considerations of blinding providers to clinically significant information such as absent or reversed EDF. The small sample size did not have the power to evaluate reversed EDF and other weaker associations with abnormal Doppler studies. For instance, 80% of patients with absent EDF had worsening severe features as a criteria for delivery but did not meet statistical significance in our sample.

Future controlled, prospective studies are warranted to evaluate the optimal frequency and timing of UA Doppler interrogation in early severe preeclampsia and to determine the interval from manifestation of abnormal waveforms (elevated, absent, reversal) to indicated delivery. Additional prospective study is also warranted evaluating the frequency of successful vaginal delivery in patients with abnormal Dopplers to counsel patients and plan for delivery. Additional studies should evaluate the association between abnormal Dopplers and maternal outcomes.

## Conclusions

Pregnancies with severe preeclampsia without FGR displayed a high incidence of abnormal UA Doppler studies associated with loss of clinical stability and adverse fetal outcomes. The groups with more impedance to umbilical artery flow tended to deliver earlier, and as the Doppler shifted from elevated S/D to absent EDF, the mode of delivery shifted to cesarean delivery with increased risk of abnormal fetal testing. These results support the utility of incorporating UA Doppler screening and surveillance into the management of early severe preeclampsia regardless of the presence of fetal growth restriction.
